# Transformation of Health and Social Care Systems—An Interdisciplinary Approach Toward a Foundational Architecture

**DOI:** 10.3389/fmed.2022.802487

**Published:** 2022-03-07

**Authors:** Bernd Blobel, Frank Oemig, Pekka Ruotsalainen, Diego M. Lopez

**Affiliations:** ^1^Medical Faculty, University of Regensburg, Regensburg, Germany; ^2^eHealth Competence Center Bavaria, Deggendorf Institute of Technology, Deggendorf, Germany; ^3^First Medical Faculty, Charles University Prague, Prague, Czechia; ^4^IT-Consulting in Healthcare, Mülheim, Germany; ^5^Faculty of Information Technology and Communication Sciences (ITC), Tampere University, Tampere, Finland; ^6^Telematics Engineering Research Group, University of Cauca, Popayan, Colombia

**Keywords:** health transformation, ecosystem, 5P medicine, architecture, knowledge representation and management, modeling, integration, interoperability

## Abstract

**Objective:**

For realizing pervasive and ubiquitous health and social care services in a safe and high quality as well as efficient and effective way, health and social care systems have to meet new organizational, methodological, and technological paradigms. The resulting ecosystems are highly complex, highly distributed, and highly dynamic, following inter-organizational and even international approaches. Even though based on international, but domain-specific models and standards, achieving interoperability between such systems integrating multiple domains managed by multiple disciplines and their individually skilled actors is cumbersome.

**Methods:**

Using the abstract presentation of any system by the universal type theory as well as universal logics and combining the resulting Barendregt Cube with parameters and the engineering approach of cognitive theories, systems theory, and good modeling best practices, this study argues for a generic reference architecture model moderating between the different perspectives and disciplines involved provide on that system. To represent architectural elements consistently, an aligned system of ontologies is used.

**Results:**

The system-oriented, architecture-centric, and ontology-based generic reference model allows for re-engineering the existing and emerging knowledge representations, models, and standards, also considering the real-world business processes and the related development process of supporting IT systems for the sake of comprehensive systems integration and interoperability. The solution enables the analysis, design, and implementation of dynamic, interoperable multi-domain systems without requesting continuous revision of existing specifications.

## Introduction

In the context of the ongoing transformations of health and social care systems to improve the safety and quality of patients' care and population health as well as the efficiency and efficacy of care delivery services under the well-known constraints, appropriate organizational and methodological paradigm changes, supported by technological innovations, are inevitable (1).

Regarding the organizational paradigm, there is a transition from organization-centric through disease-specific process-controlled care to person-centric care. This process is accompanied by technological evolutions, such as the advancement from centralized to highly distributed and mobile technologies, deploying nano-, molecular-, and bio-sensors and actuators, healthcare internet of things (IoT), also referred to as the internet of medical things (IoMT) (2), smart systems, knowledge representation, and management as well as a social business. Also, big data and analytics, learning technologies and artificial intelligence, and autonomous systems, enabled by cloud, cognitive as well as edge, and nowadays also by quantum computing, must also be mentioned here.

Regarding the methodological paradigm, care evolves from the empirical approach of general care addressing health problems with one solution fitting all through the evidence-based medicine approach of dedicated care for a stratified population with specific, clinically relevant conditions to, in combination with the aforementioned new technologies, holistic or translational medicine. Holistic medicine aims at the entirety of physical, emotional, mental, spiritual, and social wellness, focusing on prevention by fixing the underlying cause of a disease instead of improving just symptoms (3), and empowering individuals and communities (4). Translational medicine is a bi-directional, interdisciplinary concept aiming at translating biomedical discoveries into clinical benefits and stimulating research by clinical observations, frequently called the “bench-to-bedside” or “bedside-to-bench” process (5). Advancing the practice of medicine from an inexact science to precision medicine, the deployment of genomics is foundational (6). A holistic, translational medicine approach including omics disciplines, such as genomics, nutrigenomics, metabolomics, proteomics, etc., allow us to consider individual health status, genetic, environmental, occupational, and social conditions, and context (stratification of population by risk profiles), so as to understand the pathology of diseases including the individual predisposition to diseases and responsiveness to treatment. By combining all interactomes, i.e., interacting factors and components impacting health of an individual, such as genomes, epigenomes, proteomes, microbiomes, metabolomes, pharmacomes, transcriptomes, cognitive-affective behavioromes, personalized, preventive, predictive, and participative care according to the precision medicine paradigm (5P medicine) can be enabled (1, 5, 7, 8). The approach is not only deployed for the evolution of health and social care, but also for advancing the underlying scientific foundations, such as clinical studies, as mentioned already in the context of the bi-directionality of the translational medicine concept (9). Recently, Cleveland Clinic started cooperation with IBM not only to deploy its quantum computers for studying genomics, emerging pathogens, virus-related diseases, and public health threats, but also for synthesizing needed data in imaging for rare diseases, using a type of deep learning called generative adversarial networks (GANs) (10). Quantum computing also allows for new insights to understand the bindings and reactions of molecules in the design of new medications for personalized medicine (11). Another example of disruptive technologies in translational medicine is the deployment of next-generation sequencing (NGS) in clinical diagnostic and the definition of new therapeutic options at the molecular level, thereby extending and completing traditional pathological methodologies, such as histo-morphology, clinical chemistry, etc. (12).

Another term representing the described evolutionary process is “digital health.” According to the definition of the Healthcare Information and Management Systems Society (HIMSS), “Digital health connects and empowers people and populations to manage health and wellness, augmented by accessible and supportive provider teams working within flexible, integrated, interoperable, and digitally-enabled care environments that strategically leverage digital tools, technologies and services to transform care delivery” (13).

The resulting personalized, ubiquitous, pervasive, and precision health services are provided independent of time and location. Personalized pervasive health includes the individualization of diagnosis and therapy with the help of bioinformatics, genomics, but also social sciences, public health, etc. While precision medicine provides the right treatment to the right patient at the right time, precision public health can be simply viewed as providing the right intervention to the right population at the right time. By advancing the methodologies for measuring disease, pathogens, exposures, behaviors, and susceptibility, population health could advance disease prevention (14). Precision cardiology, for example, integrates diverse, wide-ranging phenotyping and genomic data on patients to better understand the mechanisms at play in inherited heart diseases, so as to support a better understanding of the links between genetic variations and clinical manifestations (15). Analyzing cellular functions, e.g., by functional proteomics is used not only to develop new immune therapies and to assess the outcome of the patients regarding disease progression, anti-tumor, or COVID-19 immunization response *via* pre- or post-treatment immune profiling but also transplants rejection based on the analysis of cellular functions, by influencing research as well as practical care (16, 17). Another example in this context is the prediction of drug resistance in melanoma cells by deploying single-cell proteomics and metabolomics to analyze melanoma cell states in response to specific stimuli (18). The tumor microenvironment (TME) comprises cancer cells, the cytokine environment, extracellular matrix, immune cell subsets, and other components (19).

Precision medicine, and the ecosystem that supports it, must embrace patient-centeredness and engagement, digital health, genomics and other molecular technologies, data sharing, and data science to be successful (20).

Furthermore, the progress of digital health tools, including mobile health apps and wearable or even implantable sensors, actively and passively collecting data and information, could help improve human health and push new approaches to the management of health conditions, thereby enhancing human data science. In that context, digital therapeutics, consumer wearables and mobile apps, connected biomedical apps, smartphone cameras, connected virtual assistants in home care, but also health system disease management apps, care teams cooperation tools, interactive programs, personal health records, telemedicine and virtual visits to the doctor, and clinical trial tools have to be mentioned (21, 22). Here, intelligent clothing using nanotube fibers to monitor heart metrics also comes into play (23).

The ability to relate data across populations requires mastering data accuracy and semantic correctness, establishing a robust data infrastructure for integration by data exchange including its verification, ultimately supporting interoperability. Thereby, the digital twin technology can also support the move to precision (and accuracy) medicine and public health (24). The paradigm changes have been frequently discussed in different documents and summarized [e.g., in (1, 25–29)].

In summary, concept-oriented, context-aware, transformed health, and social care ecosystems consider the continuum from the cell up to society or even from elementary particle to the universe. Operations of such ecosystems require communication and cooperation of principals (person, organization, device, application, component, and object) as defined by the Object Management Group (30). Those principals belong to multiple domains, including medicine, natural sciences, engineering, and also social, legal, and political sciences, and the entire systems sciences world (systems medicine, systems biology, systems pathology, etc.). They are guided by different perspectives and objectives, follow different policies, deploy different methodologies, and use different languages/terminologies. A major principle is the empowered patient and his/her social environment. Such transformed ecosystems must be inevitably integrated with appropriate security and privacy solutions, the establishment of trust, and the assurance of ethical and humanistic as well as equity, non-discrimination, and fairness principles. Table 1 summarizes the objectives of pHealth ecosystems and characteristics as well as methodologies and technologies for meeting them (28), while Table 2 aggregates technologies, methodologies, and principles for transforming healthcare ecosystems (29).

**Table 1:** The objectives and characteristics of pHealth ecosystems as well as the methodologies/technologies for meeting them (28).

**Objective**	**Characteristics**	**Methodologies/technologies**
Provision of health services everywhere anytime	• Openness • Distribution • Mobility • Pervasiveness • Ubiquity	• Wearable and implantable sensors and actuators • Pervasive sensor, actuator and network connectivity • Embedded intelligence • Context-awareness
Individualization of the system according to status, context, needs, expectations, wishes, environments, etc., of the subject of care	• Flexibility • Scalability • Cognition • Affect and Behavior • Autonomy • Adaptability • Self-organization • Subject of care involvement • Subject of care centralization	• Personal and environmental data integration and analytics • Service integration • Context-awareness • Knowledge integration • Process and decision intelligence • Presentation layer for all actors • Affective and cognition-aware computing
Integration of different actors from different disciplines/do-mains (incl. the participation/ empowerment of the subject of care), using their own languages, methodologies, terminologies, ontologies, thereby meeting any behavioral aspects, rules and regulations	• Architectural framework • End-user interoperability • Management and harmonization of multiple domains including policy domains	• Terminology and ontology management and harmonization • Knowledge harmonization • Language transformation/ translation
Usability and acceptability of pHealth solutions	• Preparedness of the individual subject of care Security, privacy and trust framework • Consumerism • Subject of care empowerment • Subject of care as manager • Information based assessment and selection of services, service quality and safety as well as trustworthiness • Lifestyle improvement and Ambient Assisted Living (AAL) services	• Tool-based ontology management • Individual terminologies • Individual ontologies • Tool-based enhancement of individual knowledge and skills • Human Centered Design of solutions • User Experience Evaluation • Trust calculation services

**Table 2:** Technologies, methodologies, and principles for transforming healthcare ecosystems (29).

• Mobile technologies, biotechnologies, nano- and molecular technologies • Big data and business analytics • Integration of analytics and apps • Assisting technologies → Robotics, autonomous systems • Natural Language Processing → Text analytics → Intelligent media analytics • Conceptualization → Knowledge representation (KR) and knowledge management (KM) → Artificial intelligence (AI) → Artificial common (general) intelligence → Intelligent autonomous systems • Security and privacy, governance, ethical challenges, Education → Asilomar AI Principles • Cloud computing, cognitive computing, social business	• Edge computing as a “family of technologies that distributes data and services where they best optimize outcomes in a growing set of connected assets” (Forrester Research) • Virtual reality and augmented reality, thereby blurring “the boundaries between the physical and digital worlds” (Gartner) • Creation of IoT-Platforms and app-ecosystems • Patient-generated health data ecosystem → multiple, dynamic policies • Web content management → Digital experience management • Databases → NoSQL technologies → Data warehouses → Graph DBs → Data lakes • EHR (including genomic data) → data exchange → semantic interoperability • Use Case Analysis → Specification → Implementation → Tooling → Testing → Certification

In the next section, we will discuss challenges and solutions for communication and cooperation between actors of transformed ecosystems, before we introduce approaches for representing and managing transformed health and social care ecosystems.

## Interoperability Challenge Under the New Organizational, Methodological, and Technological Paradigms

**Figure 1:**
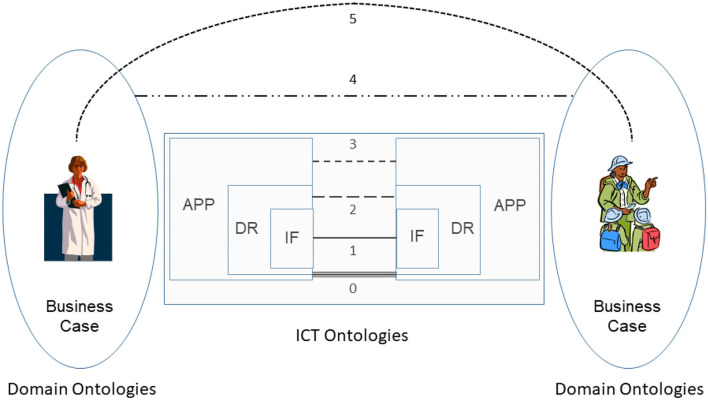
Comprehensive interoperability schema (31).

Interoperability has been traditionally addressed as an information technology (IT) challenge. As a result, the following interoperability levels from technical plug & play (0) through an interface (IF) enabled data/information exchange (1), sharing of semantics at data representation (DR) level (2) up to service sharing at application (APP) level (3) have been established [the numbers in the brackets correspond to those in the interoperability schema (Figure 1)]. The concepts and relations of the involved information and communication technology (ICT) systems components are represented using ICT ontologies.

At its core, interoperability of transformed health and social care ecosystems demands to integrate the knowledge of the business domains involved in the business case. A methodology to achieve interoperability would not be complete without taking into account human factors, such as education, skills, experiences, and social and psychological factors. In addition, commonsense knowledge must also be considered for interoperability (31). Therefore, the described advanced health and social services approach require the explicit and formalized representation of involved knowledge and skills as well as the application of pervasive, cognitive, and autonomous computing technologies for healthcare. Figure 1 presents the comprehensive interoperability challenges, where the ICT-related stuff is the simplest one.

The resulting interoperability levels are shown in Table 3, considering both the informational and the organizational perspectives.

**Table 3:** Interoperability levels of the comprehensive interoperability schema.

**Information perspective**		**Organizational perspective**
**Interoperability level**	**Instances**	**Interoperability level**
Technical interoperability (0)	Technical plug&play, signal- & protocol compatibility	Light-weight interactions
Structural interoperability (1)	Simple EDI, envelopes	Information sharing
Syntactic interoperability (1)	Messages and clinical documents with agreed upon vocabulary	
Semantic interoperability (2)	Advanced messaging with common information models and terminologies	Coordination
Organizations/Service interoperability (3)	Common business process	Agreed Cooperation
Knowledge-based interoperability (4)	Multi-domain processes	Cross-domain Cooperation
Skills-based interoperability (5)	Multi-domain individual engagement	Moderated end-user collaboration

*The numbers in the brackets correspond to those in the interoperability schema (Figure 1) (32)*.

The system represented by the subject of care and the processes of analyzing and managing his/her health comprises different levels of structural and functional complexity. The structural complexity or granularity ranges from elementary particles through atoms, molecules, cell components, cells, tissues, organs, bodies, and communities, up to population (1). Regarding the domain-specific functional, or in general, interrelational aspects of that system and its components, we have to deal with quantum-mechanical effects in the atomic and subatomic world, biochemical processes, physical interrelations throughout the continuum, social relationships in the macro-world, etc. (1). Knowledge related to those facts has been reviewed, e.g., in (25, 27–29).

**Figure 2:**
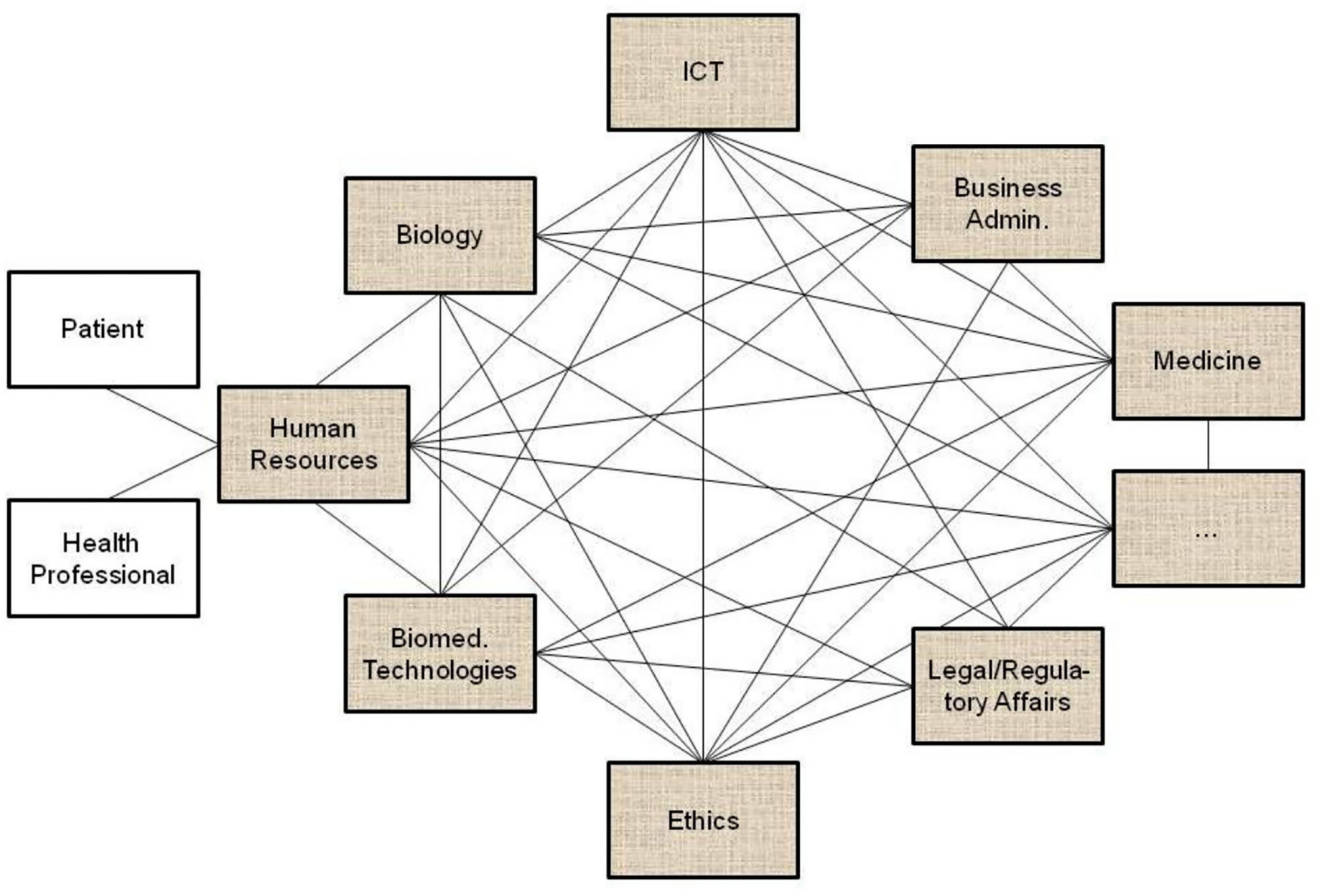
Domain-domain interoperability requesting a permanent bilateral harmonization process (the un-shaded blocks present two relevant examples of human resources in pHealth ecosystems, but there are, of course, many more).

All the domain experts involved in the aforementioned transformed health services settings describe not only the specific aspects of that system in a specific context, using their specific languages and methodologies, but also specific expression means covering natural languages, figures, equations, formulas, codes, etc. As a result, the information flow and the background knowledge of the different domains have to go through a peer-to-peer interoperability adaptation process (Figure 2). Thereby, all the existing components and their representational models and standards connected or contributing to the system (shaded in the figure) have to be newly harmonized when some components or contexts are changing, or new components have been added to the therefore highly dynamic system.

**Figure 3:**
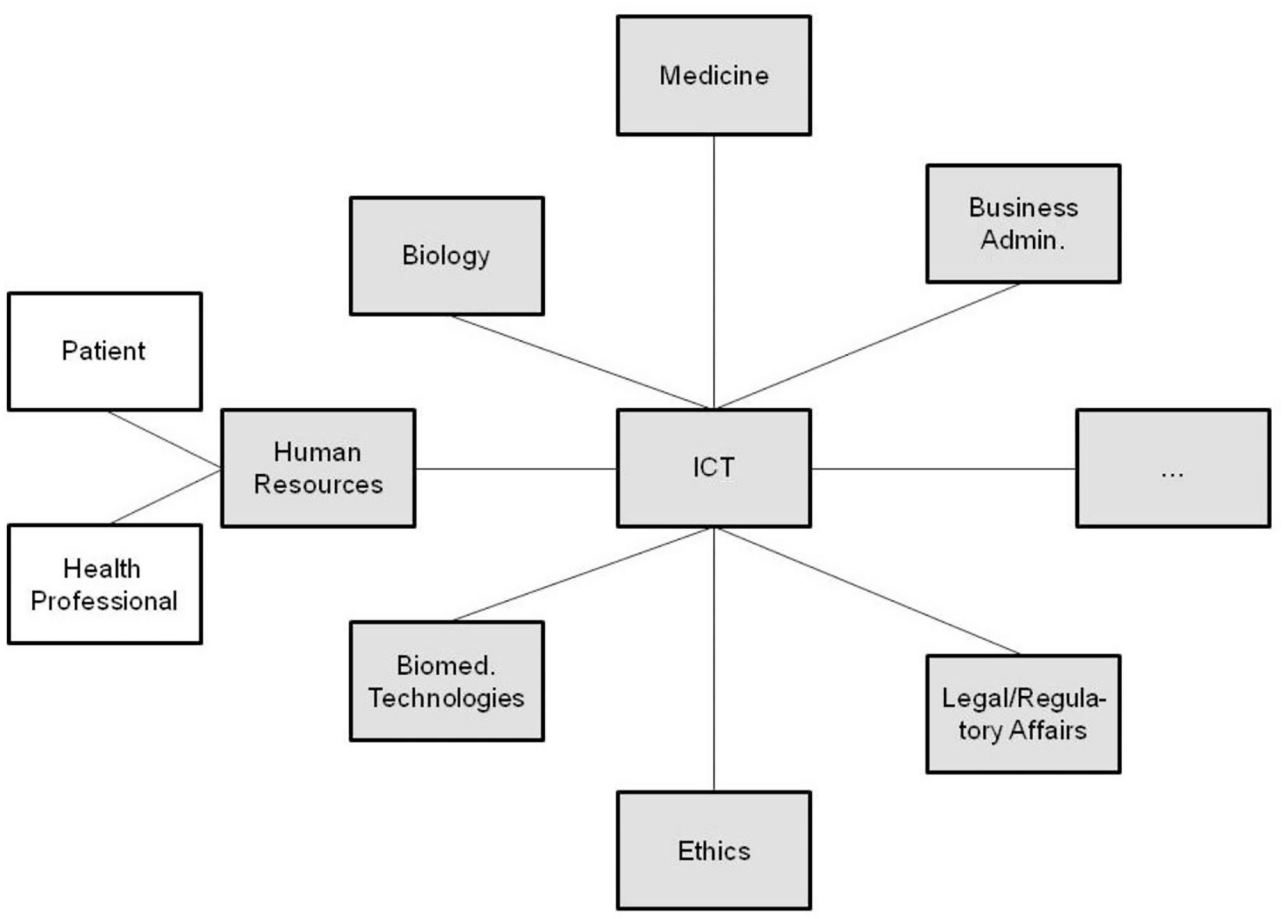
Interoperability through model and ontology domain adaptation.

An alternative approach to integrating the interrelated but different perspectives and aspects is the deployment of one domain's language, ontology, representational style, models, architectures, and standards (e.g., ICT languages, ontologies, and notations) as a reference or master all the interrelated components must be adapted to (Figure 3). Such a process is tough and demands sometimes cumbersome compromises from the parties involved. The problems faced by this approach include complexity, completeness, expressivity, and consistency of domain-specific knowledge representation languages and ontologies, which start growing when moving from implicit knowledge up to fully explicit knowledge representation, i.e., from natural language up to machine language and universal logic (32–34). While more expressive knowledge representation language and reasoning systems like traditional programming languages with their context-free grammar enable a simpler and compact expression of knowledge, they usually need more complex logic and algorithms for constructing equivalent inferences to represent transformed health ecosystems, thereby running not only into a complexity, consistency, computability and decidability, but also a completeness problem. Less expressive knowledge representation languages, such as natural languages with their context-sensitive grammar optimize restrictions to special structure vs. generative power, thereby enabling a rich and nevertheless decidable representation of real-world concepts with the support of common sense knowledge. Hence, they allow not only for an efficient representation of meaning, shared knowledge, skills, and experiences, but also facts and knowledge about a system and its domain-specific subsystems, architecture, and behavior. Therefore, many domain ontologies deploy natural-language-based domain-specific terminologies and concept representations, extensively exploited in the best practices of good modeling discussed in the Chapter “Modeling digital health systems” in this volume. More details about knowledge representation and management languages, their grammars, and relationships can be found in the study by Blobel et al. (29).

The aforementioned statements clearly demonstrate that it is impossible to represent and justify the highly complex, highly dynamic, multi-disciplinary/multi-domain transformed healthcare system by just one domain terminology/ontology or, even worse, by using ICT ontologies exemplified in the next section. The deployment of domain-specific reference ontologies representation tools furthermore excludes the addressed other domains' experts which should when thinking of the medical domain experts' role in health informatics, be in the lead, but cannot understand and deploy that environment. ICT ontologies can hardly manage dynamic systems, resulting in the permanent revision of existing components to be integrated into the system.

## Mastering the Knowledge Representation and Management Challenge in Multidisciplinary, Complex, and Dynamic Ecosystems

Focusing on different knowledge classes, such as classification-based knowledge, decision-oriented knowledge, descriptive knowledge, procedural knowledge, reasoning knowledge, or assimilative knowledge, knowledge has been defined in multiple ways (29). Davenport defines knowledge as “… information combined with experience, context, interpretation, and reflection. It is a high-value form of information that is ready to apply to decisions and actions” (35). As a result, we have to accept multiple knowledge spaces to represent the same real-world system. When representing reality according to the theory of knowledge or cognitive theory, we have to advance the cognition/sense-perception of reality toward its conceptualization (36). Doerner describes domain knowledge as reproducible and reliable models of a domain, repeatable formulated and justified in the discourse domain (discipline) by domain experts using their domain-specific methodologies, terminologies, and ontologies (37). A domain model represents that domain's perspective on reality to facilitate reasoning, inferring, or drawing conclusions. It formally describes objects, properties, relations, and interactions of a domain, enabling rational and active business in the represented domain. One important methodology to resolve the aforementioned knowledge representation problem is using domain-specific ontologies to formally represent the knowledge or concepts of each of the domains involved.

When conceptually modeling ecosystems, three levels of knowledge representation must be distinguished and consecutively processed: (a) epistemological level (domain-specific modeling), (b) notation level (formalization, concept representation), and (c) processing level (computational, implementations) (37). While the epistemological level of domain-specific modeling has been discussed so far, we will now focus on the concept of representation and formalization of the transformed health and social care ecosystem. The processing level will be considered in the Chapter “Modeling digital health systems” in this volume.

At the notation level, we need a formal knowledge/concept representation that is able to bridge between different domain-specific formal languages by uniformly representing concepts and relations of their elements. This can be done by generalizing the different ontological commitments required for those languages, i.e., the types of, and the relations between, things that the elements of the language represent (38). In model-based engineering, the Concept Representation Language has been developed to meet this challenge (38).

**Figure 4:**
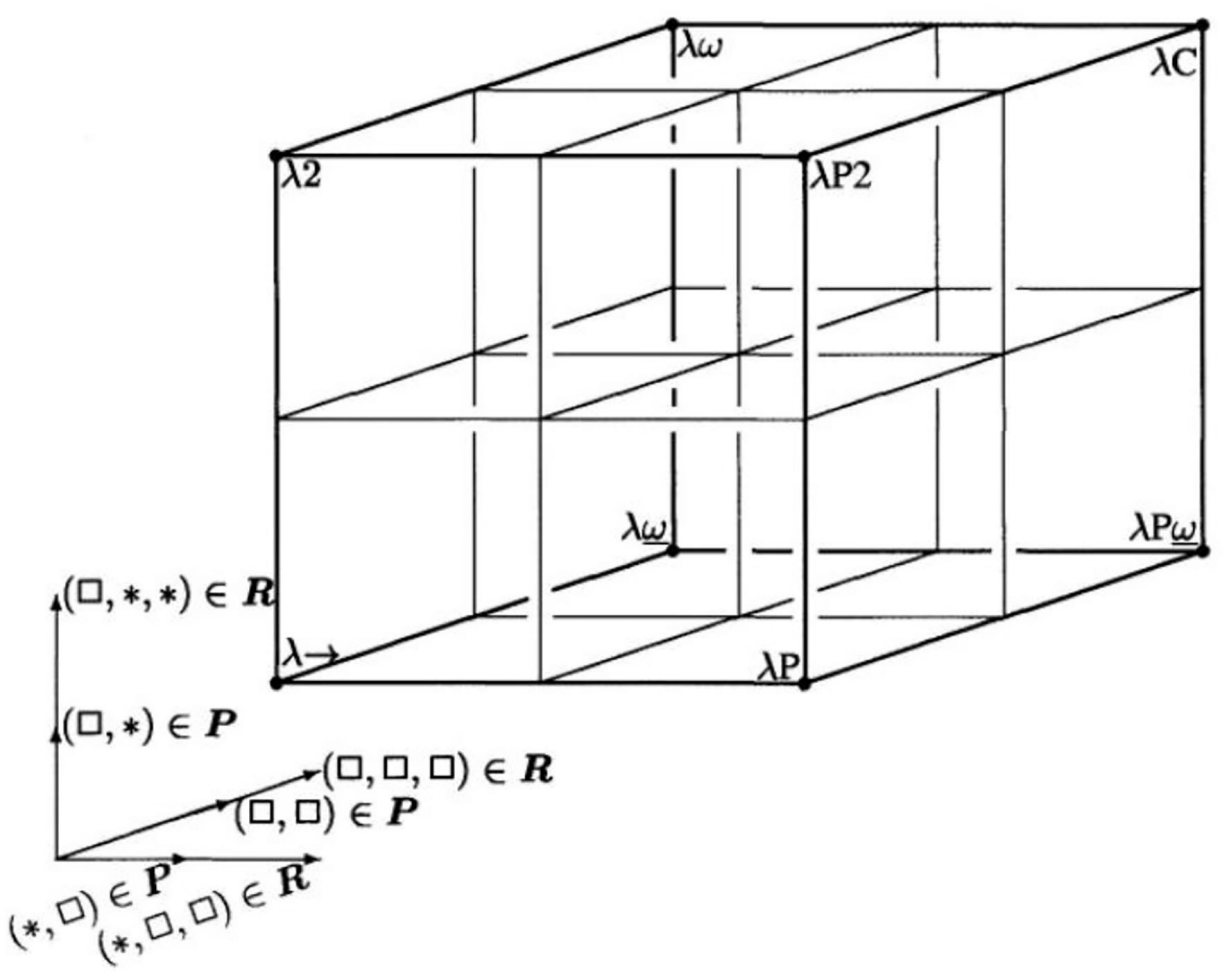
Barendregt Cube with parameters [after (42)]. *Is a generic name for an element in a series of constants, defined by Barendregt. *Represents the sorts of types in Type Systems.

In the recent past, formal logic has moved from its traditional disciplines of philosophy and mathematics to disciplines, such as computer science, cognitive science, artificial intelligence, linguistics, and several more. 25 years ago already, we developed a similar approach, not limited to ICT systems but appropriate for multidisciplinary health ecosystems, using universal type theory, originally introduced in the early years of the last century (39), and universal logics to represent any system in the universe. In mathematics, logic, and computer science, a type system is a formal system in which every term has a “type” that defines its meaning and the operations that may be performed on it (40). The advantage of type theory vs. set theory is the type theory's property of a formal language and its computability. Furthermore, it allows for the representation of any system and the relationships of its components in just one notation layer similar to the object-oriented paradigm. Both representations of the body of mathematics can be transformed into each other (41). To compare and integrate type systems, Barendregt has specified the Barendregt Cube as the combination of eight important type systems presented in a uniform way (39). For adapting other practical type systems, allowing for the grouping of sets belonging, e.g., to one domain or subsystem, the Barendregt Cube has been advanced to the Barendregt Cube with parameters, presented in Figure 4 (42).

The mathematical language of the Universal Type Theory and its representation by a Parameterized Barendregt Cube provides a proper solution for those challenges enabling to represent any formal language or informal language. To allow for the implementation of the system model for a given harmonization challenge, it might be required to go back to the most comprehensive description mode. However, for most of the scenarios, a simplified approach for representing the existing models and standards is sufficient.

For advanced interoperability of a complex, multi-disciplinary system with multiple actors performing at different skill levels, domain-specific components providing domain-specific perspectives on the system, represented using terms, concepts, and relationships of those ontologies of the domains must be structurally and functionally interlinked correctly according to the real-world system architecture. For that reason, an abstract and generic architectural model is needed that allows to represent any real-world system in any context, i.e., for any objectives, properties, perspectives, or interests bound to the considered business case and its processes. Furthermore, the definition and deployment of ontologies must be advanced through an architectural consideration of the real-world system represented to place and interrelate the ontological concepts correctly. This allows for the correct and consistent integration of different concepts of ontologies and avoids incorrect relations/equivalences of concepts provided by different domain ontologies for specific real-world elements (see also **Figure 7**). The same holds also for concept representations/models in the ICT viewpoints. As they lack contextual and implicit knowledge, simply mapping ICT concepts and models provided for different domains without considering the related granularity levels and specific contexts, unfortunately frequently practiced, is error-prone, can lead to wrong decisions and life-threatening actions.

**Figure 5:**
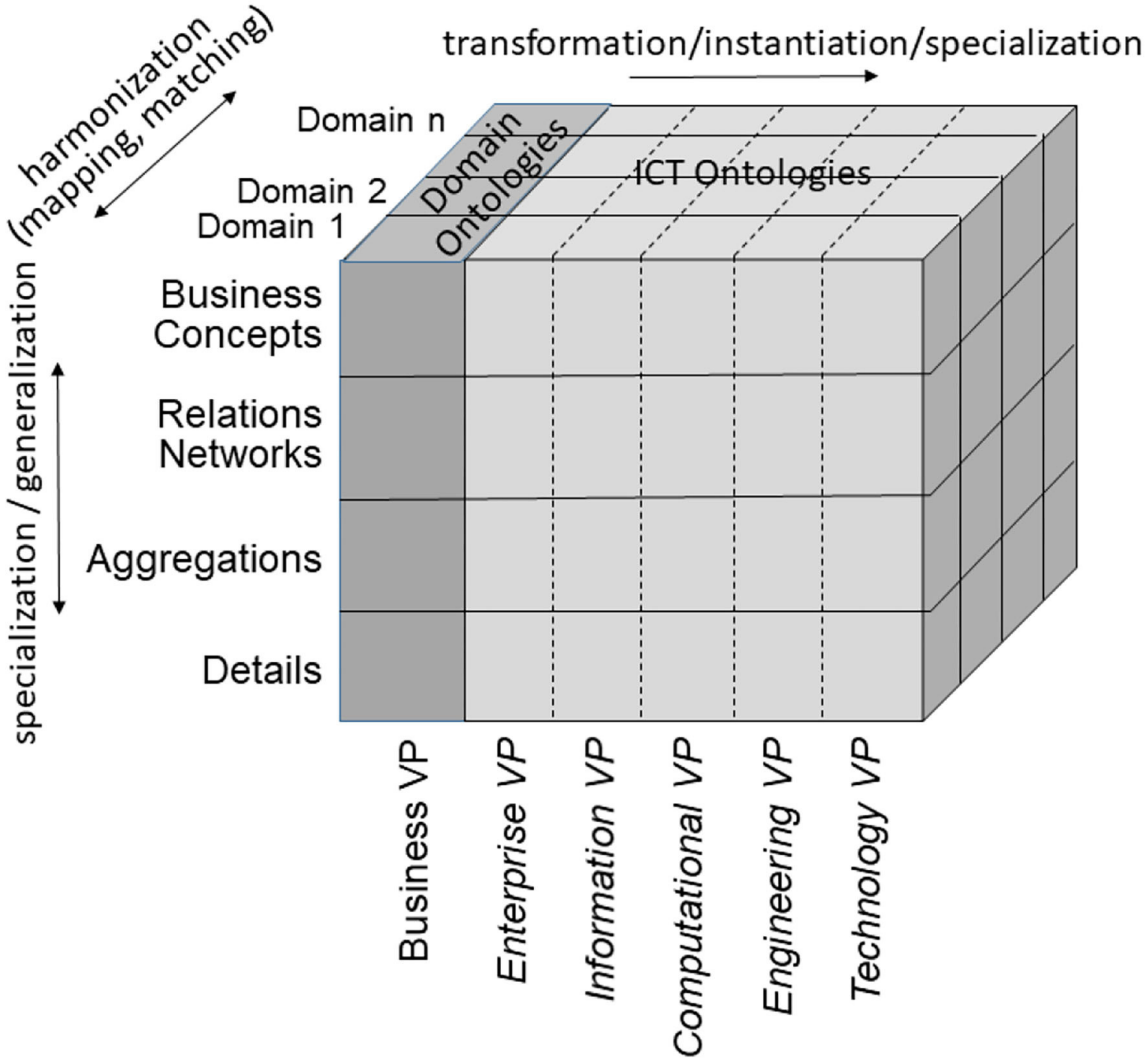
The Generic Component Model.

**Figure 6:**
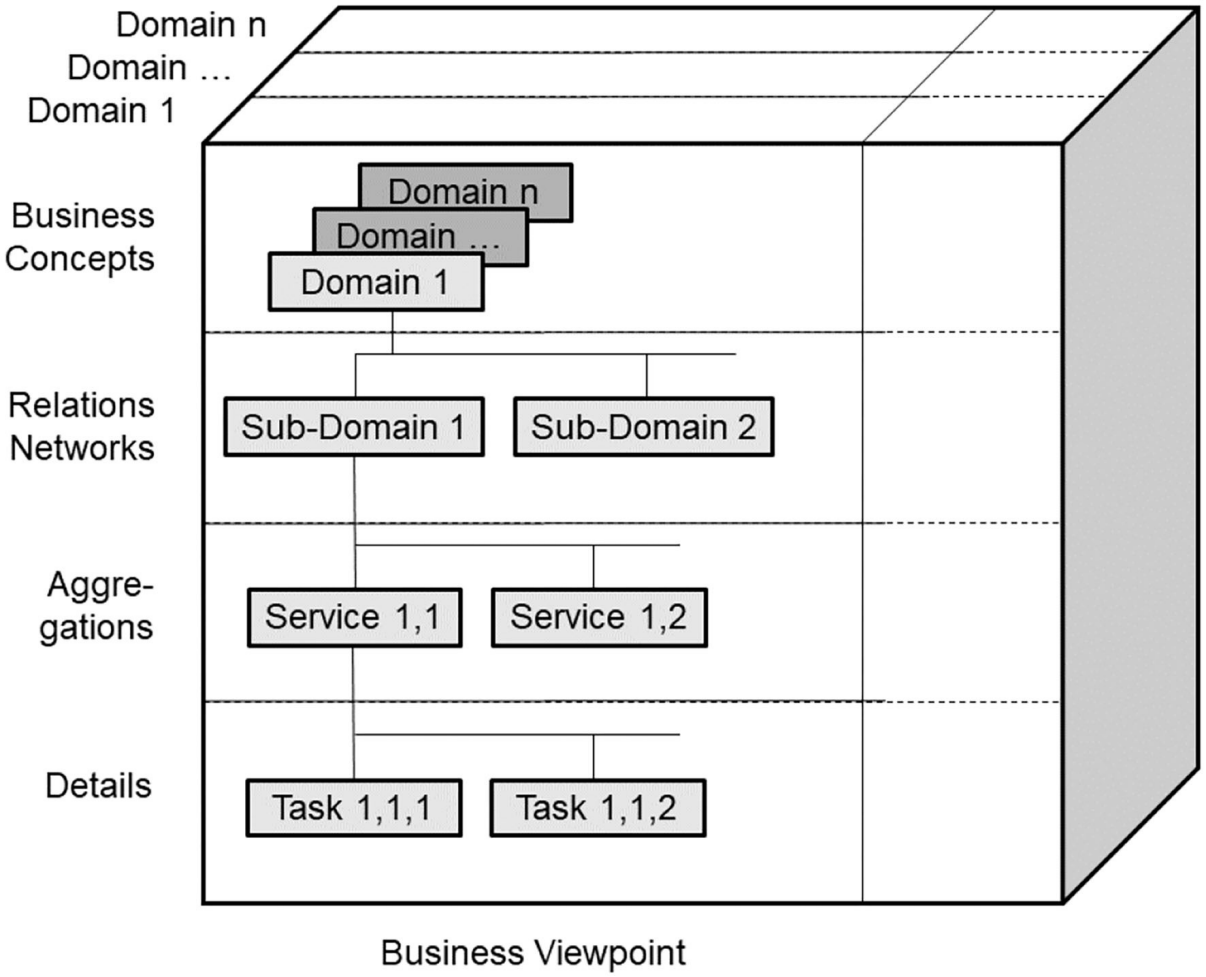
GCM granularity levels.

According to ISO 21838 (43), a domain is a collection of entities of interest to a certain community or discipline. Consequently, a domain in our approach covers specific knowledge spaces, which could be the knowledge space of a discipline or the knowledge space of an individual actor/principal. The domain ontology of the latter is not a widely agreed one, but an individual ontology. The provided ontology harmonization enables meaningful communication between specialized health professionals, frequently talking, e.g., in Latin, and laymen, using street languages (44). Meanwhile, first steps for overcoming those limitations in the ontology ecosystem by enhancing it with an architectural framework have been performed (45–47). To meet the aforementioned challenge, the mathematical representation of the Barendregt Cube has been combined with the approach of the engineering discipline of systems theory. The advantage of a systems theory approach is due to the essence of systems engineering as follows: A system groups structurally and/or functionally interrelated components, which are separated from the environment by system boundaries. Systems can be recursively defined by composing (aggregating) them to super-systems or decomposing (specializing) them to sub-systems. As systems interact with their environment, sub-systems interact with each other and with the super-systems they belong to. The challenge is to represent the architecture of a system of systems structurally and functionally. For that purpose, domain-specific epistemological models must be generalized by transforming them into a universal knowledge representation (KR) notation, which has to be validated on the real-world system and thereafter adopted, if needed (37). Meanwhile, the approach is internationally acknowledged as ISO 23903:2021 “Interoperability and Integration Reference Architecture - Model and Framework” (48), standardizing the Generic Component Model (GCM) (49–53). It presents any real-world system using three dimensions (Figure 5):

a) the decomposition (composition) of the system in (of) its components (subcomponents), etc. (Figure 6);
b) the perspectives or the aspects of that system, represented by the domains addressing those perspectives/aspects, using the domain-specific ontologies (Figure 6);
c) the evolution of the system, in the context of digital health, the development process of implementing the system in an ICT environment following, but extending, the ISO 10746 ODP-RM (54) or the Rational Unified Process (55), respectively.

The GCM is a top-level architectural model and framework of a system of systems, formally describing the system components, their functions, and interrelations structurally and behaviorally, thereby representing specific aspects (domains) by related subsystems. For each business case, the subsystem components, their functions, and interrelations are instantiated by naming and representing them using the specific ontologies of the domains involved in that business case. For enabling this representation of a real-world system by its ICT-independent domain ontologies, the GCM provides a Business Viewpoint additionally to the five ODP-RM viewpoints. For the other viewpoints, ISO 10746 defines ICT-specific languages and representation styles, such as Business Process Modeling Language (BPML) and Business Process Modeling Notation (BPMN) (56), the Unified Modeling Language UML (57), or programming languages. For healthcare-specific aspects, healthcare-specific ICT ontologies standardized in ISO 13606 (58), ISO 12967 (59), ISO 13940 (60), openEHR Archetypes (61), ISO 13972 (62) or the outcome of the HL7® Clinical Information Modeling Initiative (CIMI) (63), and also implementable specifications following the HL7® RIM ontology (64), such as HL7®V3 (65), and nowadays HL7® FHIR® resources (66, 67) are widely deployed.

As we can consistently model and compute only systems of reasonable complexity, the system analysis or design has to address partial systems when considering higher granularity levels of the system in question. The architectural dimension of system component composition/decomposition, combined with the recursivity of the approach, allows for describing the continuum of systems from elementary particles to the universe in a generalized and standardized way. By considering just that detail of the continuum needed for managing the business objectives, like an amplifier glass magnifies just that part of the continuum the glass focuses on, so that the aforementioned complexity problem is overcome. At all levels of the complexity of the system, the GCM defines the same generic granularity levels: business concepts, relations networks, aggregations, and details (Figure 6). The business concepts represent the conceptual domains of the system involved in the business case. The relations networks represent the subdomains within each domain. The aggregations level represents the services and concepts within a subdomain. The details describe the actions/tasks making up the services.

In the Business Viewpoint, the GCM domains are represented by the use of domain-specific ontologies. However, in order to ensure that all domain specific ontologies are consistently organized and as far as possible are future proof, they all need to be derived from an over-arching domain-neutral ontology representing the architecture of a real-world system in question from an abstract system-theoretical perspective. In that way, the domain-specific ontologies representing the domain-specific aspects of the system can be correctly and consistently integrated (mapped, matched), nevertheless reflecting all the domain-specific knowledge available. The resulting model can be easily transformed into corresponding ICT concepts.

**Figure 7:**
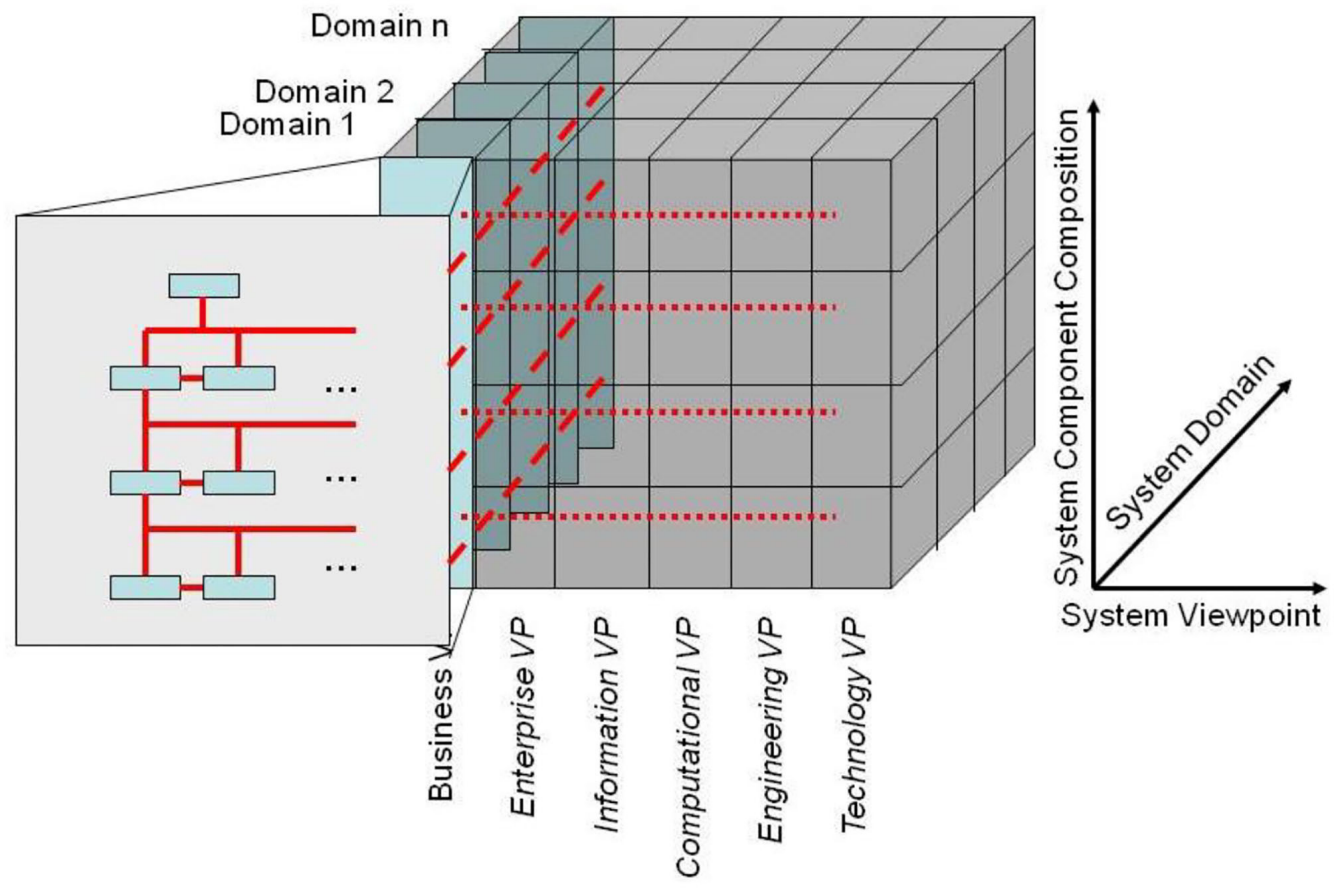
The GCM model and framework.

The GCM (or ISO 23903) framework describes how to use the GCM in interoperability and integration settings. For properly representing the structure and behavior of the system, only components at the same granularity level can be interrelated, thereby reflecting the constraints ruling the interrelations of the components within (System Component Composition/Decomposition Dimension) and between the involved domains (System Domain Dimension) (as shown in Figure 7). For mapping components at different architectural granularity levels, they must be generalized or specialized first to comply with the mandatory framework. The same holds also for the systems development process through different viewpoints.

**Figure 8:**
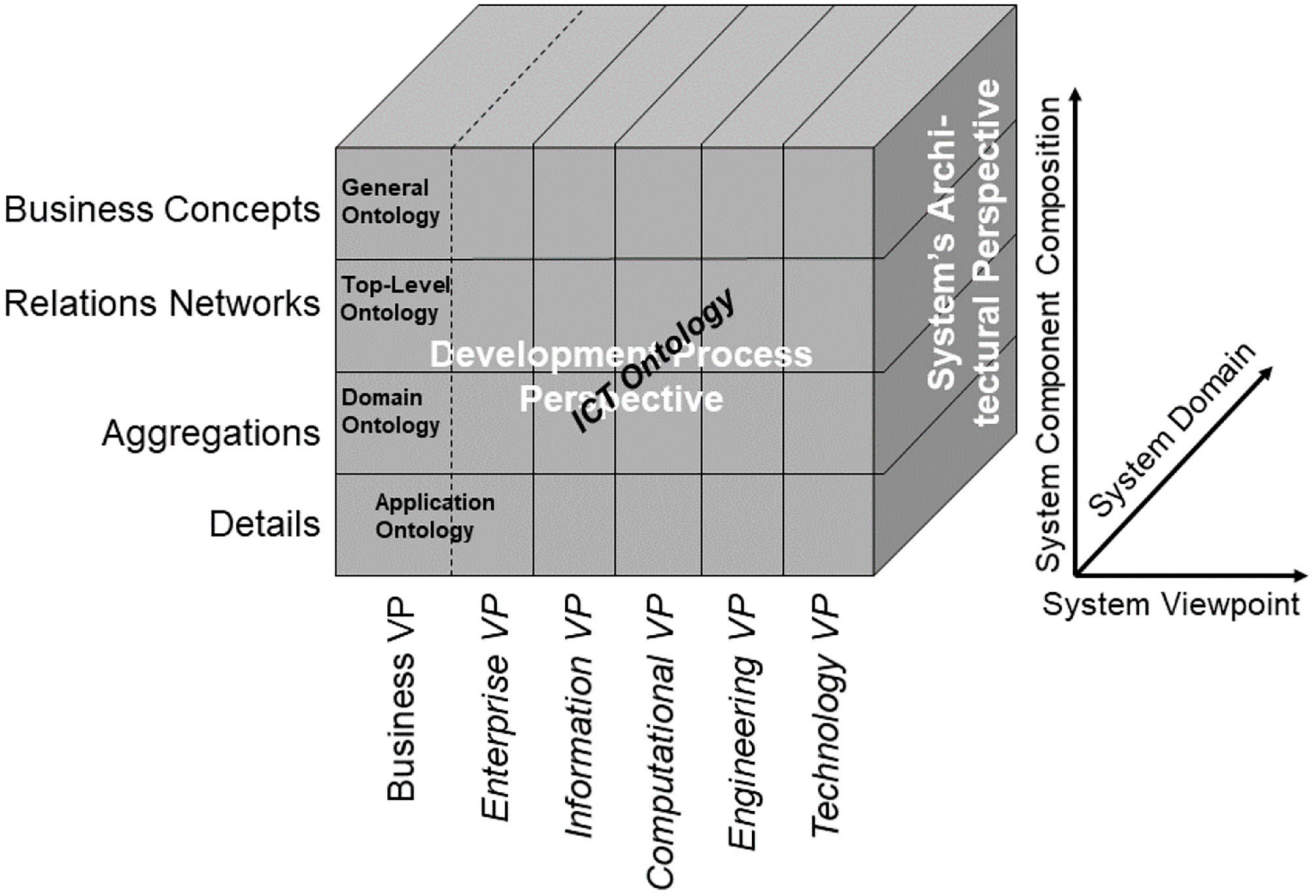
Managing the system of ontologies using the GCM.

As demonstrated, the GCM can also be used to advance basic sciences, such as the development and engineering of domain-specific ontologies and their relations to other concept representations. As aforementioned generic ontology, a top-level ontology according to ISO/IEC 21838, following the Basic Formal Ontology (BFO) from the Open Biological and Biomedical Ontology (OBO) Foundry, should be deployed (43). Figure 8 presents the system of ontologies deploying the GCM.

## Interoperability and Integration in Ecosystems Mediated by the GCM Reference Architecture

**Figure 9:**
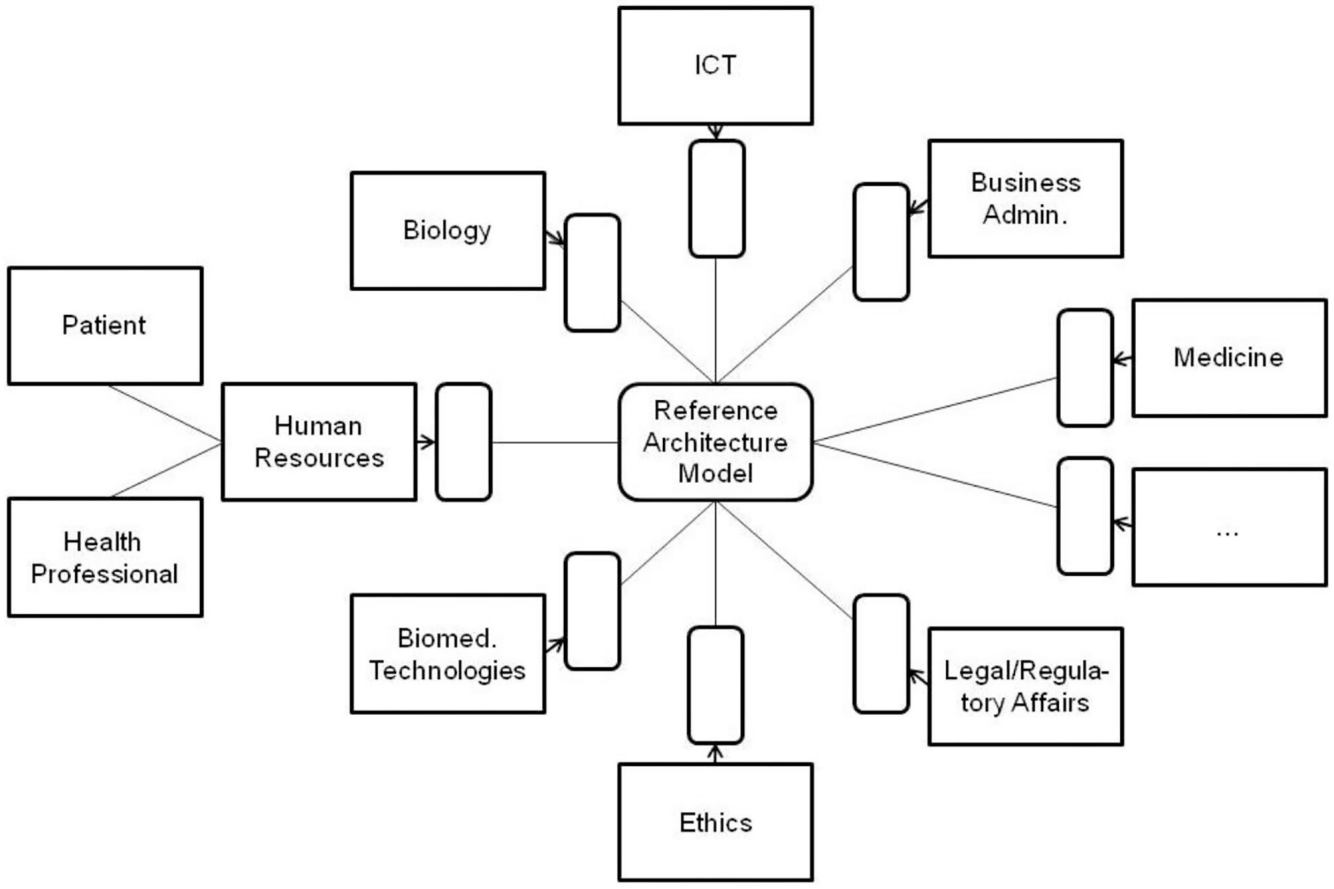
Interoperability mediated by the GCM reference architecture.

The solution for meeting the described business objectives and challenges of the emerging health services paradigms and overcoming the aforementioned problems is the definition of a formally represented, system-oriented, ontology-based, policy-driven reference architecture model, and framework any component or domain-specific subsystem can adapt to. Such an approach allows for mapping different knowledge spaces, different representation styles, different maturity levels, etc., thus providing not only interoperability between and integration of different domains including different individual skills levels, but also different specifications without prior revision, thereby clearly qualifying it against solely ICT-level interoperability and integration efforts. This way, it can design and manage collaboration and cooperation of multidisciplinary systems including living and non-living principals. For solving ICT interoperability challenges, standard data interfaces or application programming interfaces (API) have been specified and implemented. While this approach was defining the structure and semantics of data to be exchanged between independently developed applications, in our solution, the structure and behavior of a system and its representation have to be specified. As the data and related applications in the information exchange paradigm remained unchanged, the existing models and standards remain unchanged in the interoperability and integration approach of the comprehensive systems as well. The domain-specific subsystems to be integrated have just to be re-engineered once by correctly placing, representing, and interrelating their components in the GCM model according to the GCM framework to allow for interoperability- and integration-enabling harmonization (Figure 9) (48).

Thereby, constraining relationships have to be defined in a formalized way when not yet specified in the underlying ontologies deployed. For specific-use cases and specific models, it might be necessary that the model to be integrated must be refined to represent all the required GCM components.

**Figure 10:**
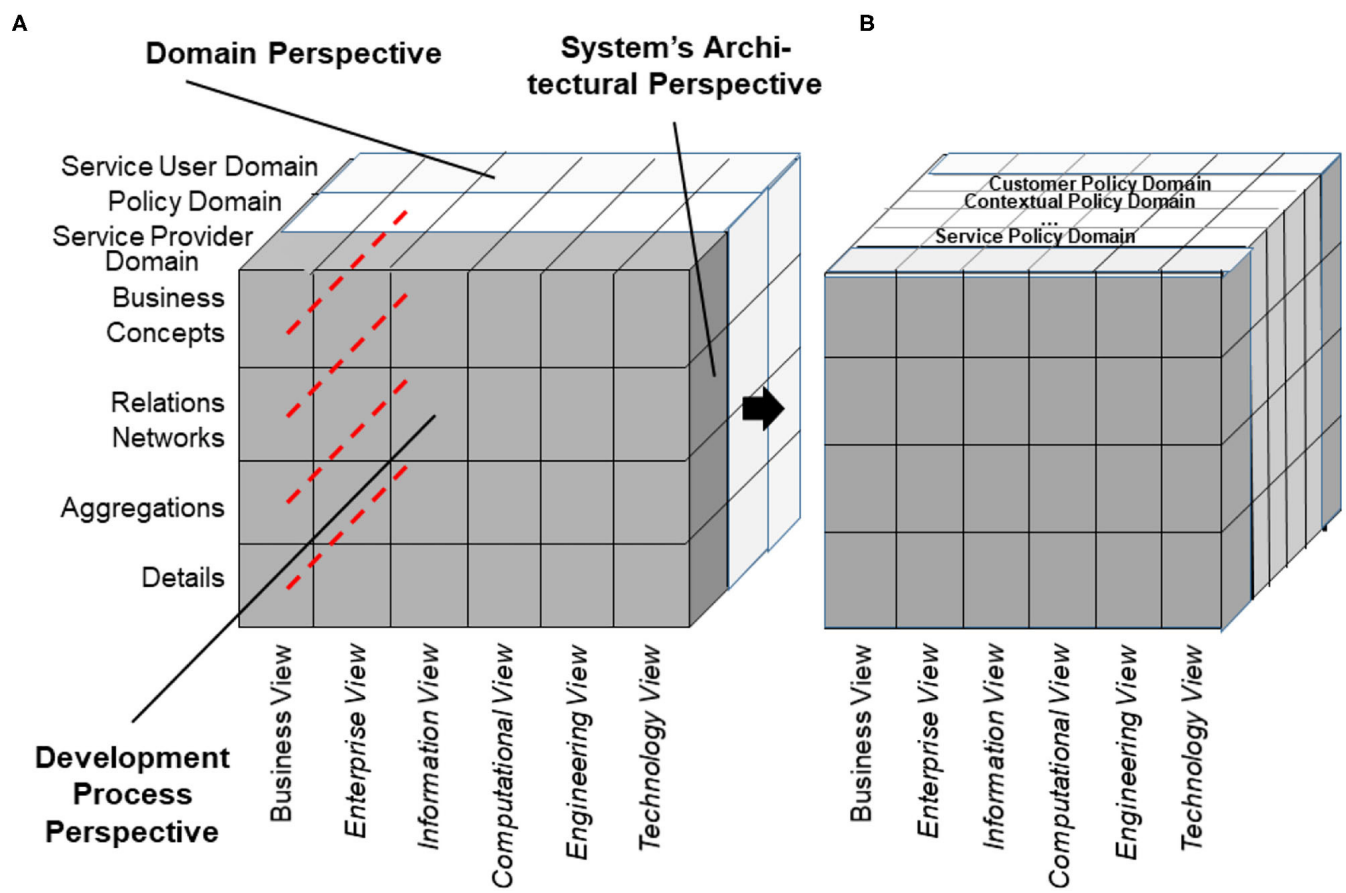
Architectural representation of the policy domain **(A)** and its specializations **(B)**.

A special domain is the policy domain, ruling and constraining the relations between the subsystems, thereby controlling the behavior of the system and also impacting its acceptance and usability. According to ISO 22600:2014 (68), a policy is a set of legal, political, organizational, functional, and technical obligations for communication and cooperation. The policy domain must be refined into policy-subdomains deploying specific ontologies. Among others, not only the individual's expectations and wishes (customer/user policy domain), security, privacy, trustworthiness, but also the ethical and legal concept spaces (contextual policy domain), and procedural requirements, such as the best medical practices (service policy domain), have to be mentioned here (Figure 10).

## Practical Deployment of the GCM Model and Framework

The GCM model and framework according to ISO 23903 has been widely implemented to enable interoperability between, and integration of, models, standards and solutions mainly in the health and social care domain. Some examples are shortly introduced as follows.

**Figure 11:**
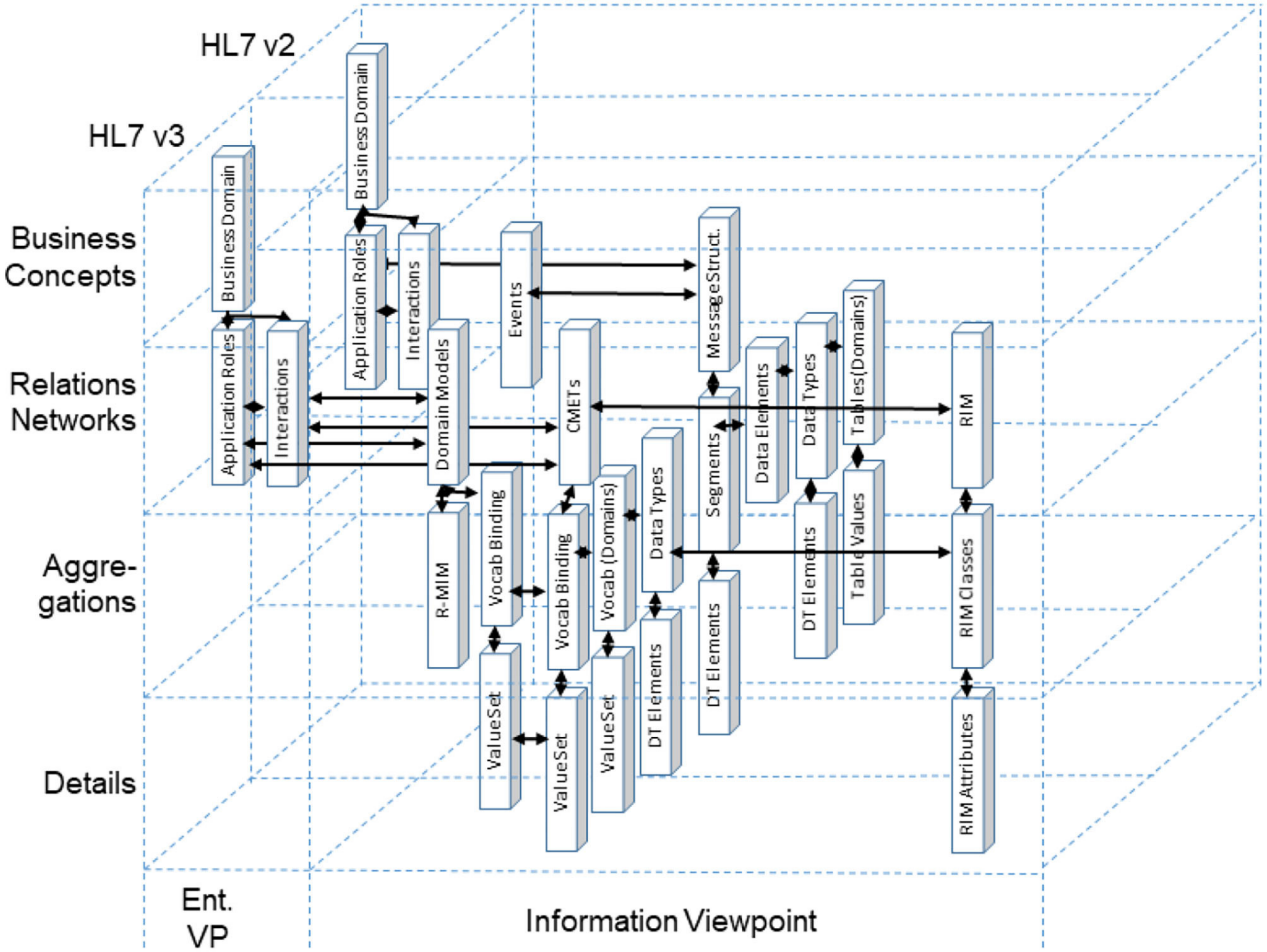
Re-engineering HL7 v2 and HL7 v3 using the GCM reference architecture model.

Based on the higher-level protocol specification of the University of California at San Francisco (UCSF), the globally most important data exchange standard in health settings, referring to Level 7 of the ISO/OSI protocol, is the Health Level 7 (HL7) standard HL7 v1, released in 1987 in the USA for testing, followed by HL7 v2.x for production in 1990 (64). Both standards define *ad hoc* specifications of data elements, data types, and messages implemented to exchange administrative, financial, and clinical information in the form of text messages. The *ad hoc* approach was advanced to the conceptual, model-driven approach of HL7 V3 with its HL7 V3 Development Framework (HDF) and its health information ontology defined in the HL7 Reference Information Model (RIM), standardized in ISO/HL7 21731 (63). For easing or even enabling the integration, both still applied specifications have to be architecturally and conceptually re-engineered, as demonstrated in Figure 11 (69–71).

**Figure 12:**
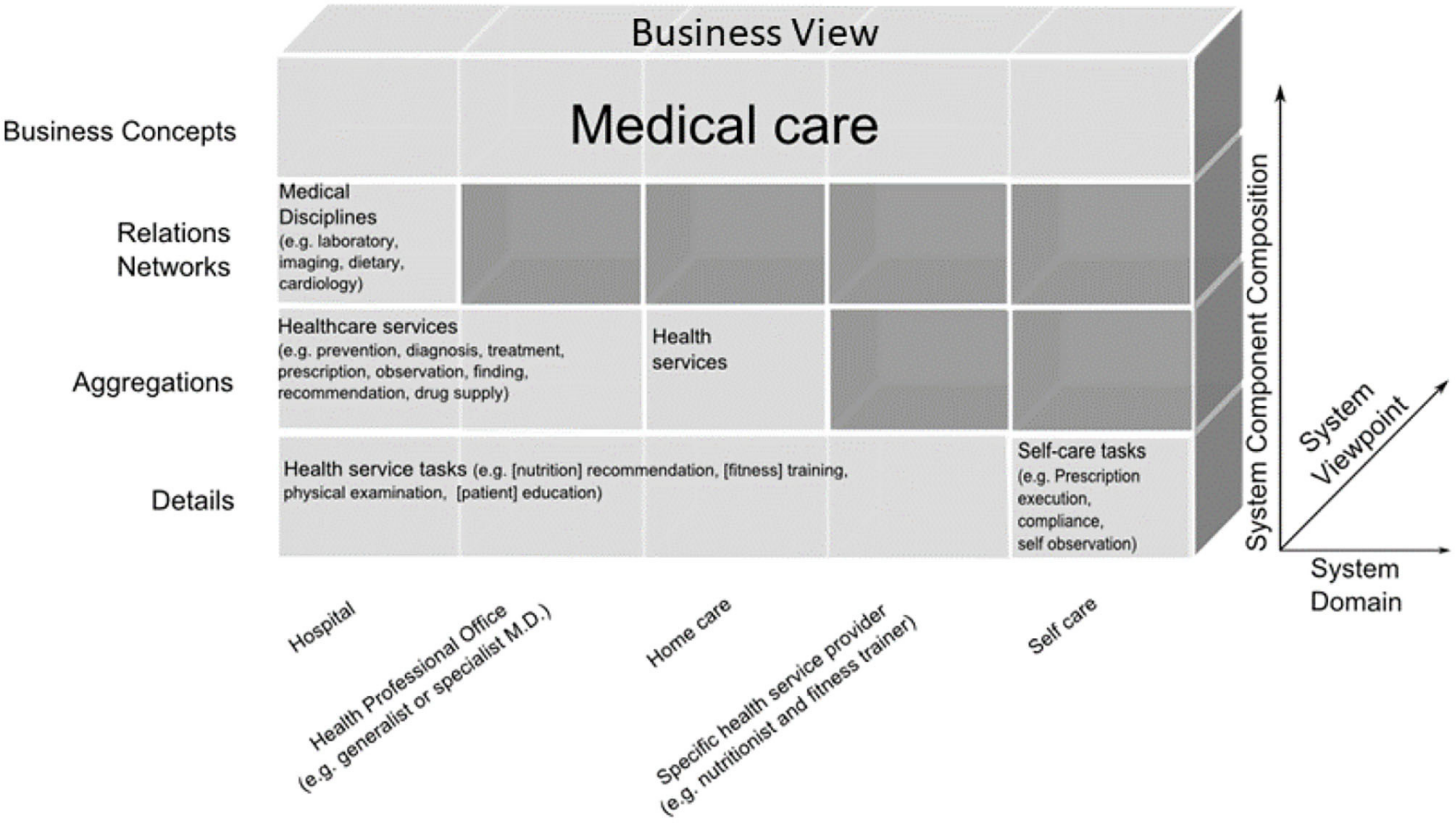
T2D domains in the GCM business view representation (67).

Another example is the automated development of interoperable Web services for Type 2 Diabetes (T2D) Care Settings including primary, secondary, and tertiary care, home care and self-engagement, dieticians, etc., based on the standardized approach (72–75) (Figure 12).

**Figure 13:**
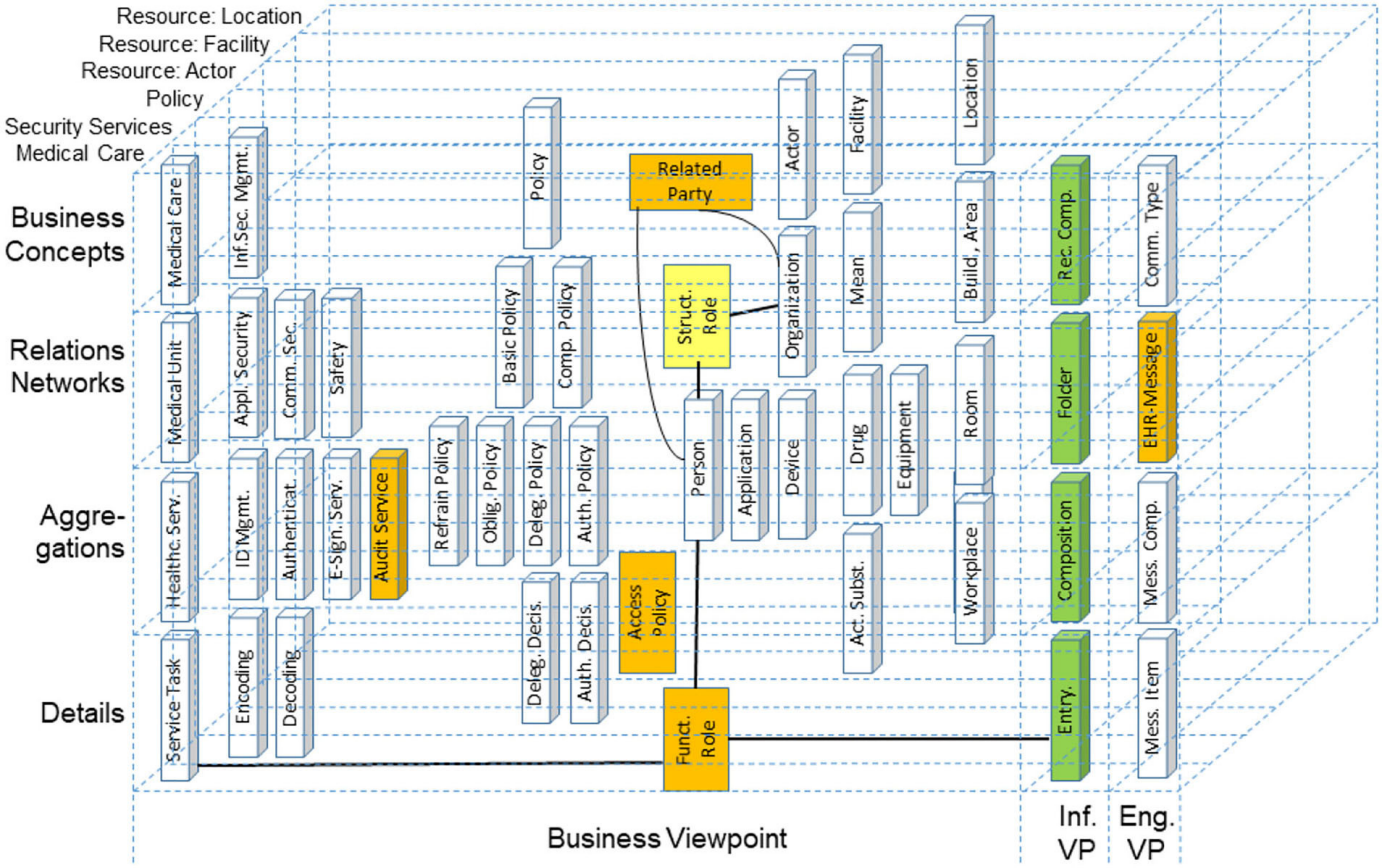
Reengineering the ISO 13606-1 reference model in the context of the HL7® composite security and privacy domain analysis model.

Many standards are dedicated to a specific topic or subdomain, such as technical specifications (devices, components) or specific technologies. Healthcare is by nature, interdisciplinary. This especially counts not only for security and privacy issues considering legal, social, ethical, and procedural issues, but also for individual perceptions, wishes, and expectations. Therefore, the GCM approach was first deployed in security and privacy standards for health, such as ISO 22600, ISO 21298 (76), or the HL7® Composite Security and Privacy Domain Analysis Model (77), recently replaced by the HL7/ANSI Security and Privacy Logical Data Model (78), and also in standards integrating security and privacy aspects in their solution, such as ISO 13606 (Figure 13).

**Figure 14:**
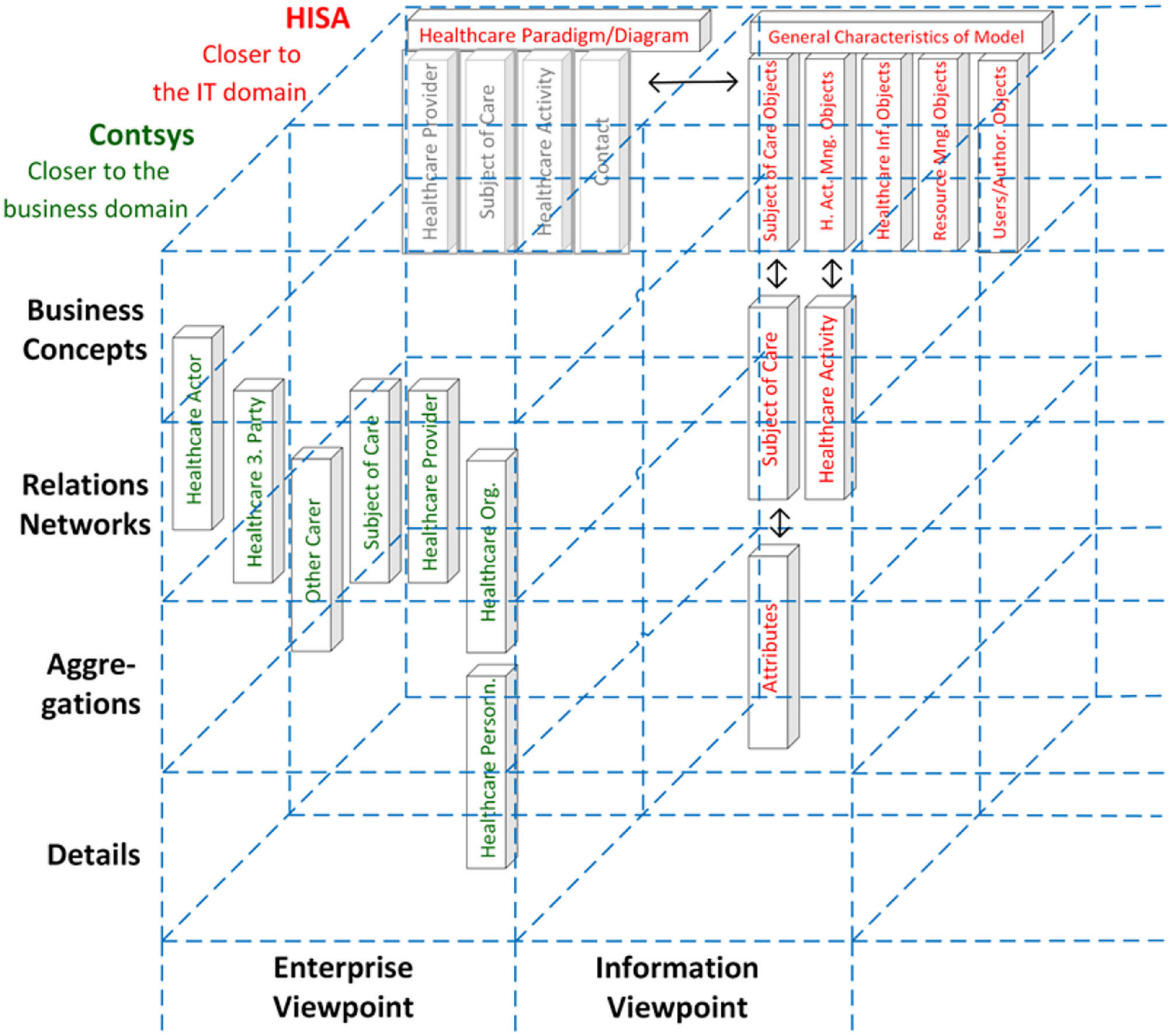
Harmonization of concepts from ISO 12967 (HISA, presented in red) and ISO 13940 (Contsys, presented in green) (43).

The turn to transformed health and social care ecosystems and related standards increasingly required integrating work products from different Standards Developing Organizations (SDOs). This fact on the one side and the establishment of ISO 23903 on the other side resulted in the inclusion of the GCM model and framework in most of the ISO/TC 215 Health Informatics standards addressing more than one sub-domain for meeting their challenges. The latter is exemplified with the harmonization of concepts from ISO 12967 (HISA) and ISO 13940 (Contsys) (Figure 14).

An example of using the GCM for ontology management to ensure semantic interoperability between different EHR systems is demonstrated in the study by Adel et al. (79).

## Practical Use of the GCM Model and Framework in the Information Modeling Process

**Figure 15:**
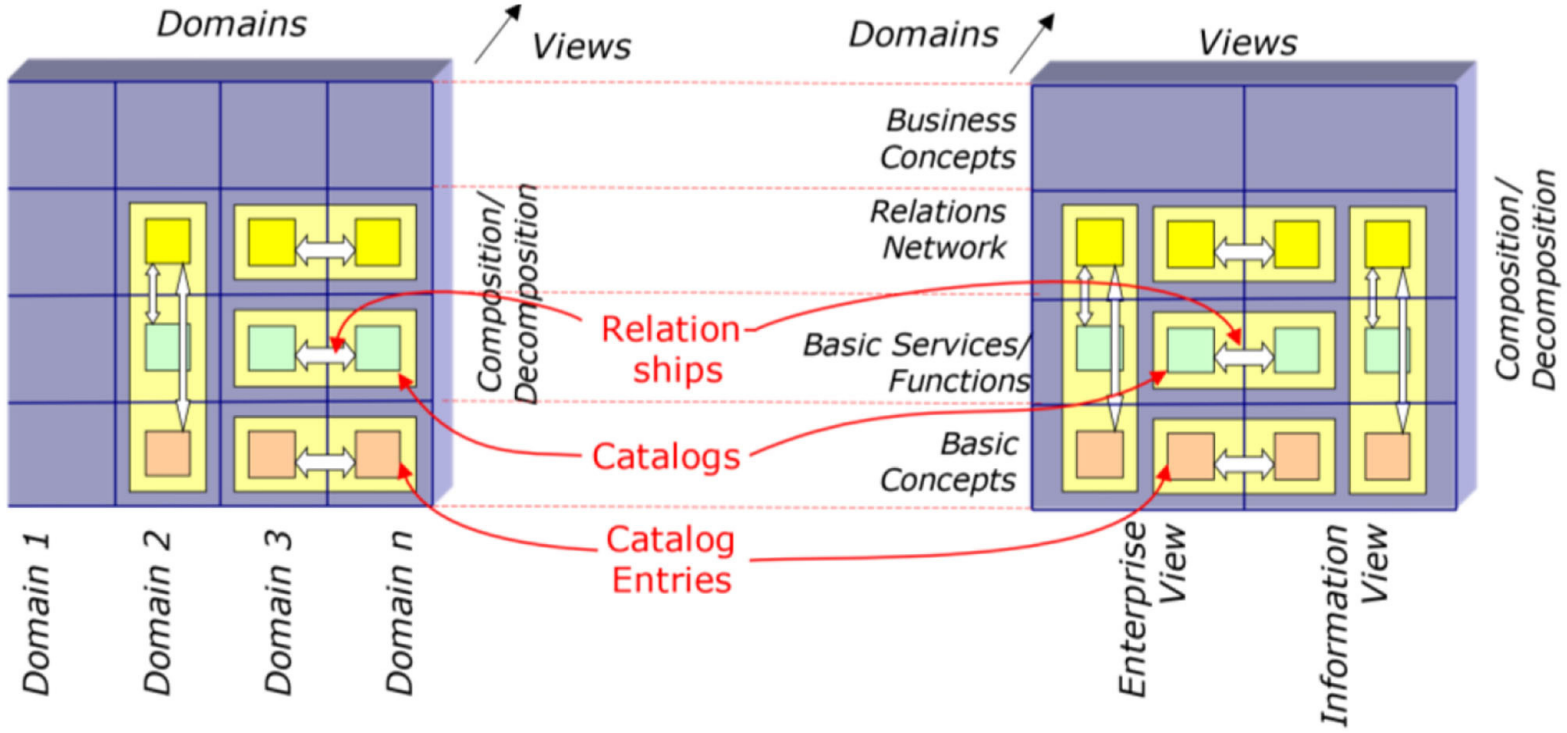
Bridging different domains or views (65).

Figure 11 exemplifies the different information objects that are used within two example communication standards. According to the methodology provided by the GCM framework, relationships can only be established either horizontally or vertically, but not in a diagonal direction (Figure 15).

**Figure 16:**
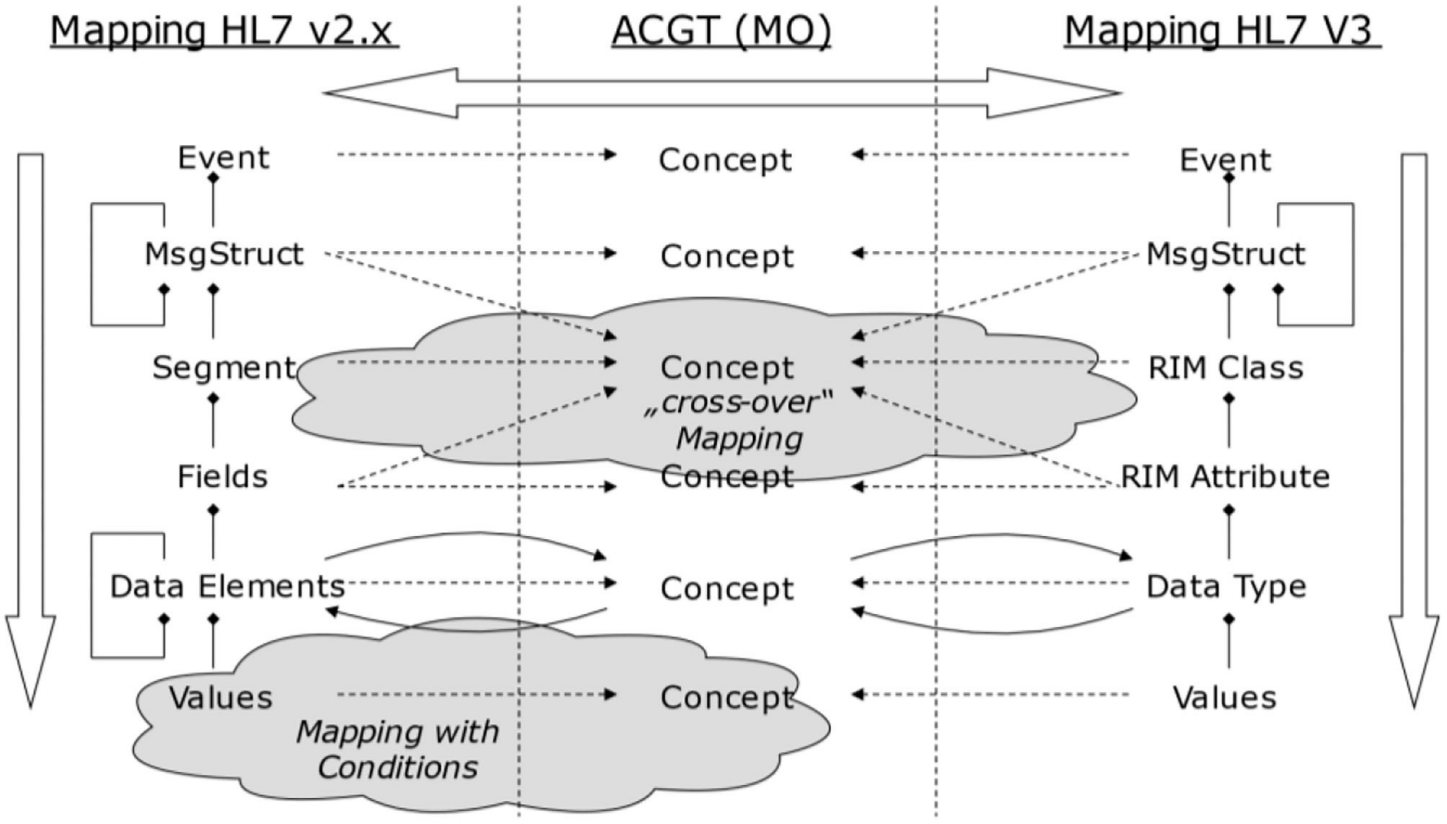
Bridging different domains with the help of a mediator domain (71).

Following the GCM framework, we can only instantiate a GCM architecture for domains and components contributing to the considered business system use case. Therefore, we can only interrelate model or specification components that have a dedicated and semantically clear relationship. For communication standards, this mechanism can be used to map them as ICT ontology to an application domain (ontology), and therefore bridge them accordingly. Of course, because of the diverse semantics of the objects, this cannot be done directly but with the help of a mediator domain. In Figure 16, ACGT, the Advancing Clinico-Genomic Clinical Trials on Cancer Master Ontology is used for this purpose (71), but other application domain ontologies would work as well.

From a practical perspective, information modeling starts with platform-independent domain-specific information models, ideally facilitating BPMN or other formal languages, which are supported by graphical representations (tools) to help with an understanding by domain specialists. In a second step, such a model can be converted into an ontology-based representation form that allows for computational support to check consistency or completeness. Once that is in place, a correct bridging, either manually or semi-supported by tools, can start.

An important aspect is an alignment with a formal ontology like BFO. This ensures that wrong mappings can be detected by reasoners. For example, a mapping from an event to observation can be brought forward for manual inspection. Completeness, a second aspect, can be verified in this way as well.

Table 4 compares the different modeling paradigms.

**Table 4:** Comparing data model levels, dimensions of modeling, data model at different information level, and the ISO interoperability and integration reference architecture model, applied to specification examples (22).

**Data model level**	**Dimen-sion of modeling**	**Data models at different information levels**	**Modeling actors**	**Model scope**	**ISO 23903 interop. & integration RA**	**Examples**		
Very-high-level data model	Know-ledge space	External	Business domains stakeholders	Scope, requirements and related basic concepts of business case	Business View			ISO 23903 Interoperability and Integration Reference Architecture
High-level data model	Know-ledge	Conceptual	Business domains stakeholders	Relevant information and representation & relationships of basic concepts	Enterprise View	DCM, CSO		
							ISO 10746 ODP-RM	
Logical data model	Information	Logical	Data modelers and analysts	Layout & types of data and object relationships	Infor-mation View	HL7 V3 (CMETs), HL7 CIMI, openEHR Arche-types, FHIM		
					Compu-tational View	HL7 FHIR		
Physical data model	Data	Physical	Data modelers and developers	Implementation-related and platform-specific aspects	Engineer-ing View			

## Discussion and Conclusion

Collaboration is a challenge to meeting business objectives, and interoperability is a vital capability to achieving such collaboration. Therefore, it is not first a matter of the ICT domain, but one of the user domain. Interoperability requires sharing of knowledge and skills, which should be built on a hierarchical system of ontologies. Multi-disciplinary interoperability solutions interrelating life sciences, natural sciences, technology, legal and social sciences, etc., require an architecture-centric systems approach to the domains of discourse represented by their ontologies, thus enabling the formalization of representation and integration of the systems including correct ontology mapping. Based on the mathematical representation of the universe using the Universal Type Theory in combination with system-theoretical approaches, the GCM has been developed in the nineties and evolved to a reference architecture model. It not only allows for harmonizing/mapping of models and standards without requiring their revision or change, but also helps in understanding how, where, and why diverse specifications are different, or what their advantages/disadvantages are. So, not only just different specifications but also different versions of one specific specification or standard can be mapped. The approach enables both the analysis and design of complex, multi-disciplinary (multi-domain) systems, thereby meeting the challenges of advanced organizational, methodological, and technological paradigms for health and social services delivery (80).

The system-oriented, architecture-centric, ontology-based, policy-driven approach to transforming health and social care ecosystems integrates different domains and communities, thereby bridging the gap between different languages, representation styles, and skills. Therefore, the solution is foundational for managing our increasingly complex and dynamic reality, possibly helping to stop endless and fruitless discussions about why one specification should be preferred above the other. The approach presented in this paper, has been exemplified for health and social care, but can naturally be deployed in any other domains.

The aforementioned technologies and domain challenges will be addressed in specific papers in this volume, dedicated to those aspects.

## Data Availability Statement

The original contributions presented in the study are included in the article. Further inquiries can be directed to the corresponding author.

## Author Contributions

BB planned and designed the paper and authored its major part. FO authored the section “Practical Use of the GCM Model and Framework in the Information Modeling Process”. All authors contributed to the article and approved the submitted version.

## Conflict of Interest

The authors declare that the research was conducted in the absence of any commercial or financial relationships that could be construed as a potential conflict of interest.

## Publisher's Note

All claims expressed in this article are solely those of the authors and do not necessarily represent those of their affiliated organizations, or those of the publisher, the editors and the reviewers. Any product that may be evaluated in this article, or claim that may be made by its manufacturer, is not guaranteed or endorsed by the publisher.
